# Red Lines in the Ocean: Sea Routes on Early Modern East Asian Maps

**DOI:** 10.1080/03085694.2023.2281136

**Published:** 2024-02-29

**Authors:** Elke Papelitzky

**Affiliations:** Department of Culture Studies and Oriental Languages (IKOS), University of Oslo

On many early modern Japanese maps of Japan, a network of lines surrounds the archipelago. These lines designate routes one could take from one harbour to the other. Parallels to this practice also exist in early modern Chinese and Korean maps, making statements about networks, connections, and possible travel paths on sea. Drawing routes over the ocean is a strange thing to do. In contrast to roads on land, there are no physical remains of a route on the sea. When weather or environmental conditions change, ships can chart a different course. Roderich Ptak therefore suggests that we should think of sailing routes as ‘mental constructs.’^1^ By drawing lines on a map, mapmakers make statements that concretize this ‘mental construct,’ fixing the route in relation to specific geographic locations, defined by islands and other waypoints.

The oldest extant dated map of Japan already shows sea routes. This 1306 map, which is one of the oldest extant maps with sea route lines worldwide, depicts a schematic representation of the Japanese provinces. Routes branching out from the capital region around Kyoto are marked in red, prominently on land, but a line also connects Shikoku with Honshu.^2^ In doing so, the islands are visually shown to belong together, linked to the seat of the emperor.

From the seventeenth century onward, mapmakers not only visually connected the major islands of the archipelago, but also indicated routes between cities and harbors. Commercially successful maps such as those by Ishikawa Ryūsen 石川流宣 mark such routes (fig. 1).^3^ In addition to drawing lines, these maps mark the distance for each segment in *ri* 里 (One Japanese *ri* is slightly less than 4 km). Ryūsen also added notes on distances of land-based travel between cities at the margins of his maps. As in the case of earlier maps, combining routes on land and sea effectively places them into the same conceptual category, resulting in a comprehensive network where it did not matter if one travelled by sea or by land and which, in Marcia Yonemoto’s words, ‘makes movement—both actual and imagined—possible.’^4^ Maps of Japan with routes were extremely common in early modern Japan and map readers certainly were familiar with this image of their country.

**Fig. 1.**
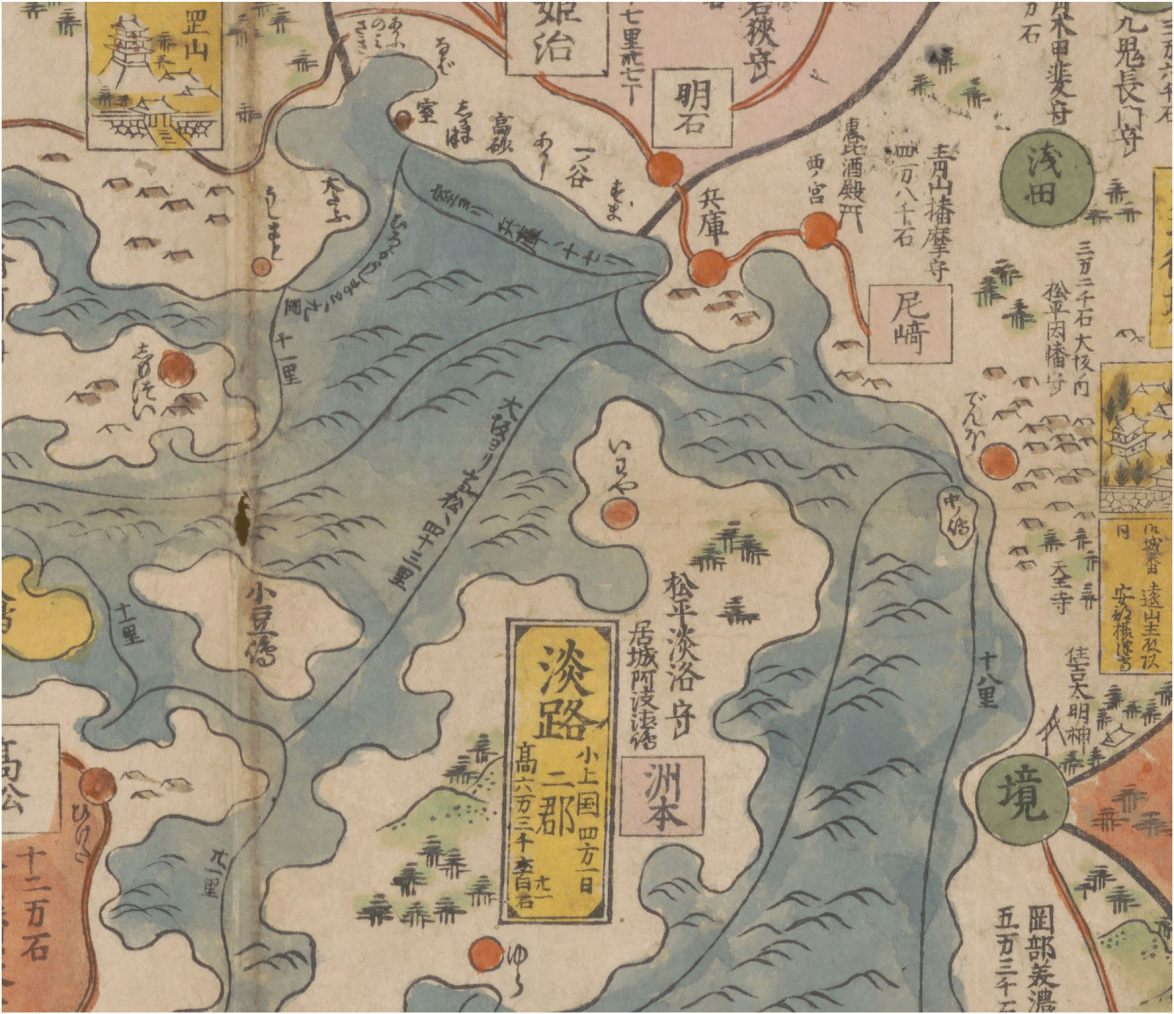
Detail of Ishikawa Ryūsen’s *Nihon kaisan chōrikuzu* 日本海山潮陸圖 (Map of the sea and mountains, tides and lands of Japan), 1691 edition published by Sagamiya Tahē 相模屋太平, Library of Congress (LOC), Geography and Map Division, G7960 1691 .I7. Hand-coloured woodblock print on paper. 81 × 171 cm. The section shows the southern coast of Honshu Island around Osaka Bay, including the north-eastern tip of Shikoku and Awaji Island. Route lines connect harbours and islands and are annotated with the distance in *ri* for each segment. Image in the public domain, courtesy of the LOC.

Few Chinese and Korean maps mark sea routes along the Chinese and Korean coast respectively. The few examples we have, both in the case of China and Korea, tend to emphasise one single route that meanders between islands without making statements about harbours along the way (fig. 2).^5^ The line becomes a visual representation of only the major maritime route, as indicated in the preface of one of the Korean maps: ‘Red lines indicate major routes on land or sea.’^6^ Due to geography, a traveller in China (and to some extent in Korea) needed to step on an ocean-going ship less frequently than a traveller in Japan. Major cities in China and Korea could be found both at the coast and inland, while Japan’s archipelagic geography and arboreous and mountainous inland required more travel by sea. It therefore does not surprise that we find more maps with sea routes of the Japanese islands but fewer of the coasts of China and Korea.

**Fig. 2.**
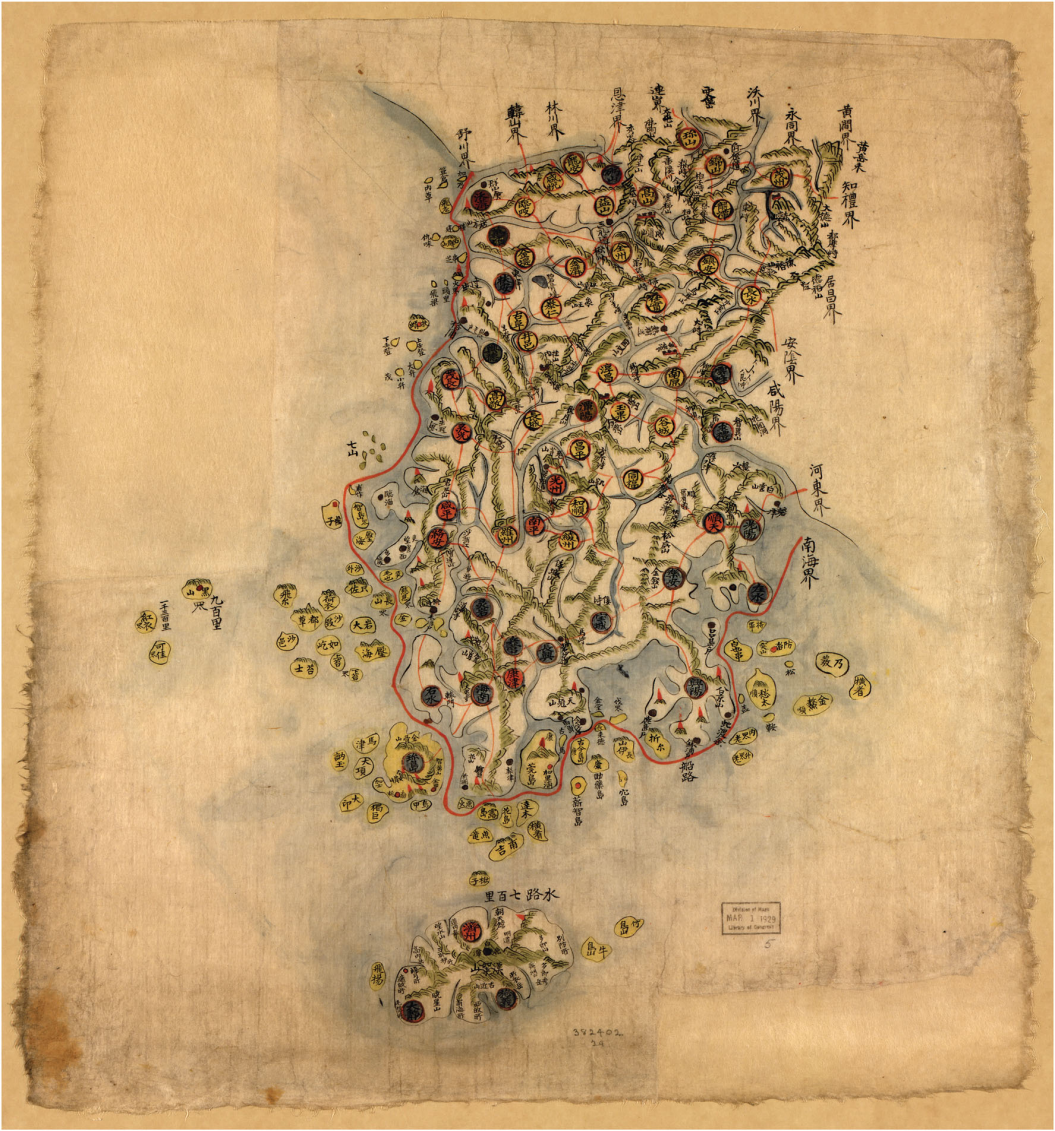
Manuscript map of Kangwŏn Province from an anonymous, untitled atlas, eighteenth or nineteenth century, Library of Congress, Geography and Map Division, G2330 .T6 176-. 67 × 47 cm. One single red line surrounds the coast of Kangwŏn province, passing between several islands off the coast. Cheju island is depicted in the south. A note mentions that the sea route to it measures 700 *ri* but the island is not graphically connected to the mainland with a line. Image in the public domain, courtesy of the LOC.

Chinese maps that indicate routes tend to use a more global approach. Instead of connecting harbours, cities, and islands within the country, they focus on connecting other countries with China. The so-called Zheng He Map (extant in two early-seventeenth-century books) is one prominent example. It relates to the early-fifteenth-century voyages to the Indian Ocean by Zheng He 鄭和 and maps the coasts his fleet visited, marking routes as dotted lines, and adding information on compass bearings and time needed to travel along these routes using the vocabulary of seafarers.^7^ This map reflects Ming China’s expansive connections with the Indian Ocean world.

The lines on these maps connecting China with other regions in Asia could represent a variety of functions. They could highlight tribute relations as in the case of the sixteenth- to eighteenth-century maps depicting the route between Fuzhou and the capital of the Ryukyu kingdom, Naha.^8^ Routes could also illustrate military matters: A mid-sixteenth-century map shows routes from Japan which so-called Japanese pirates had used to attack the Chinese coasts, helping the readers to visualise the pirate threat that was rampant in that period (fig. 3). The routes could also reflect on private trade, as for example the early-seventeenth-century Selden Map does, highlighting Fujianese trading networks in Southeast Asian waters.^9^ In short, there are route maps illustrating basically every aspect of early modern China’s connections with Asia.

**Fig. 3.**
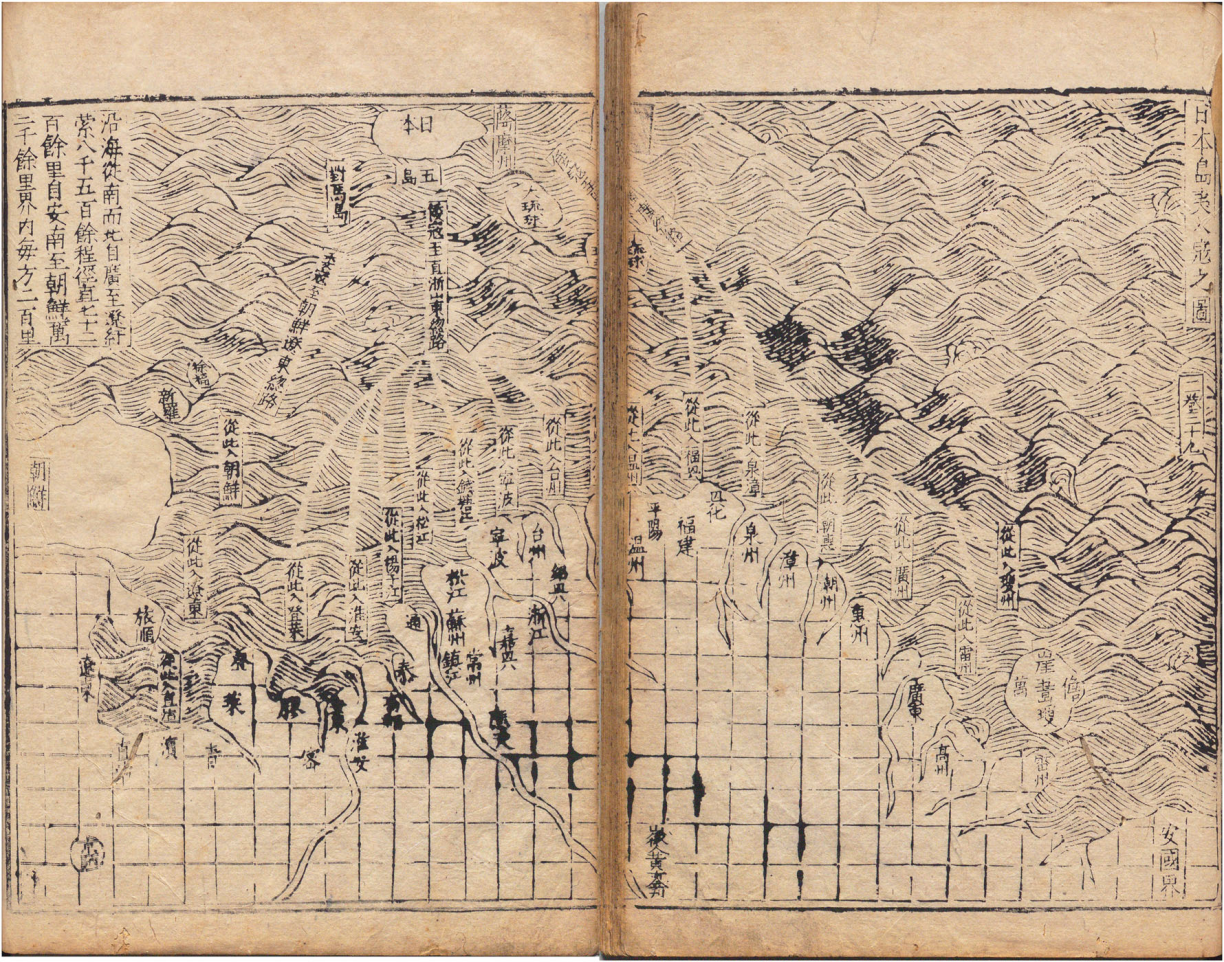
‘Riben daoyi rukou zhi tu’ 日本島夷入寇之圖 (Map of the Japanese island barbarians coming to invade [China]) from Zheng Ruozeng’s 鄭若曾 *Chouhai tubian* 籌海圖編 (Illustrated naval strategy, 1562), National Archives of Japan, 史198–0011. Woodblock print. 25.5 × 35.5 cm. The map shows the coast of China at the bottom and one island labelled as Japan at the top. Narrow strips are left blank in a sea of waves designating the attack routes of the Japanese pirates. The routes are labelled: ‘From here they enter [city name].’ Image in the public domain, courtesy of the National Archives of Japan.

Maps with route lines also looked beyond Asia, integrating the Americas, Europe, and Africa, places very few East Asians at the time would see with their own eyes. In Japan, such maps appeared on screens in the late sixteenth century.^10^ These expensive objects made for the ruling elite draw lines in red connecting Japan with Europe and the Americas. Often, the screens come paired with a map of Japan and, as such, they show Japan’s place in an expanding world. The red lines strengthen this narrative even further by marking the connections explicitly.

More systematic mapping of trans-oceanic routes on printed maps started to be widespread from the late-eighteenth century. Mapmakers such as Zhuang Tingfu 莊廷旉 in China and Shiba Kōkan 司馬江漢 in Japan created world maps in two hemispheres.^11^ The sources of the Japanese and Chinese mapmakers were European maps that had included track lines and Kōkan and his contemporaries copied these routes on their own maps. However, most East Asian maps remove the names of the explorers, fundamentally changing the message of the lines.^12^ Instead of highlighting past achievements of explorers, the lines show a network of routes, connecting East Asia with the whole globe.

When these map images travelled between places, the function of the route lines could change. Within East Asia, the lines stayed consistent. An early-eighteenth-century Korean map of Japan made no change to the route lines when copying from a Japanese map.^13^ Similarly, when the Koreans Ch’oe Han-gi 崔漢綺 and Kim Chŏngho 金正浩 used Zhuang Tingfu’s world map to create their own in 1834, they retained the lines as on the Chinese map (Fig. 4).^14^ When maps travelled in the other direction, from Japan to the Low Countries, the lines were erased. European maps of Japan followed Japanese maps for most of the outlines, but none of the known printed European maps of Japan includes even a hint of the routes so ubiquitous on Japanese maps of Japan.^15^ While routes showing the achievements of past European explorers were highly relevant to European world maps, lines emphasising an integrated Japanese archipelago were not, just as the names of European explorers did not mean anything to East Asian contemporaries.

**Fig. 4.**
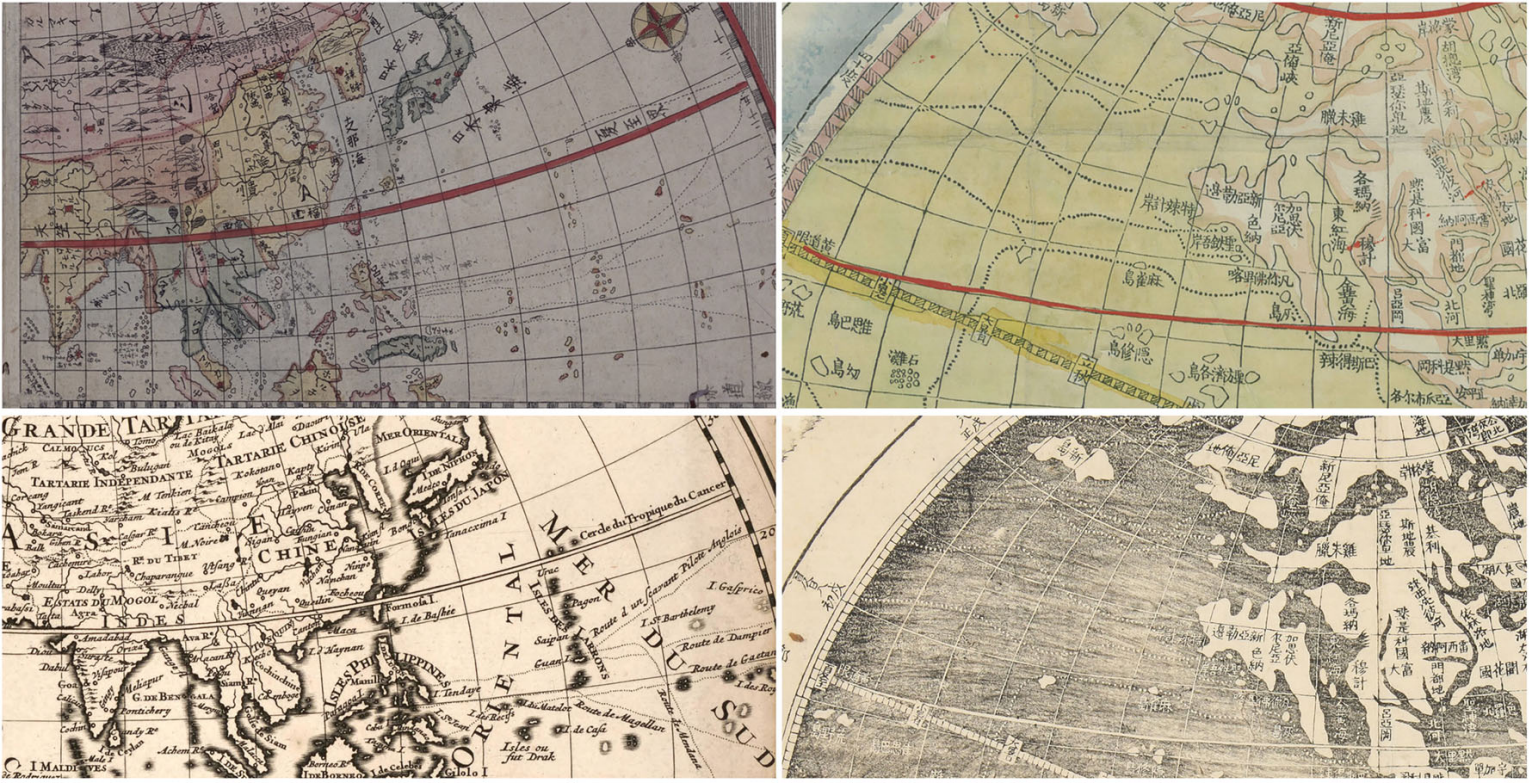
Clockwise from upper left: Shiba Kōkan, *Chikyū zenzu* 地球全圖 (Complete map of the globe), 1792, Main Library, Kyoto University, 6-05/シ/1貴別; Zhuang Tingfu, *Da Qing tongshu zhigong wanguo jingwei diqiushi fangyu gujin tu* 大清統屬職貢萬國經緯地球式方輿古今圖 (Terrestrial map of then and now of the myriad countries belonging and bringing tribute to the Great Qing in the form of a globe with longitudes and latitudes), 1800, Bibliothèque nationale de France, Cartes et Plans, GE C-5354 (RES); Ch'oe Han-gi and Kim Chŏngho, [world map], 1834, Seoul National University, Kyujanggak Institute for Korean Studies, 古4709-15; Library of Congress, Geography and Map Division, Nicolas Sanson, *Mappe-monde dressé sur les observations de mrs. de l'Academie royale des sciences et quelques autres et sur les memoires les plus recens*, c. 1696, Library of Congress, Geography and Map Division, G3200 1696 .S3. Dotted lines cross the oceans on all four maps, but while on Sanson’s map the lines are labelled with information of explorers who travelled these routes, no such information is recorded on the three Asian maps. All images in the public domain.

Mapmakers within East Asia shared an understanding of what functions the route lines could assume. East Asian maps rarely highlight past, concretely travelled routes. Instead, they focus on providing information for future real or mental travel. More importantly, showing a present network was the preferred function of routes on East Asian maps, regardless of what they were connecting. This view of the world, which had its roots in the seventeenth century and then became popular in the eighteenth century, exhibits a clear narrative desire to be part of the global world, already before the mid-nineteenth century Opium Wars and the Meiji Restoration that are so often considered a turning point for a re-orientation of East Asia towards the ‘West.’ The maps speak of a self-understanding that emphasises maritime integration and not isolation.

## NOTES AND REFERENCES

1 Roderich Ptak, ‘Sailing Routes as Mental Constructs: Preliminary Notes on Their Semantic Dimensions and Assumed Functions,’ *Journal of Asian History* 52, no. 2 (2018): 235–57.

2 The map is held by Ninna Temple in Kyoto. For a reproduction, see Nakamura Hiroshi, *Nihon kochizu taisei*, new edition (Tokyo: Kōdansha, 1974), plate 1. The only earlier map with sea routes I am aware of is a mid-thirteenth-century Chinese map of China engraved on a stone stele titled *Yudi tu* 輿地圖, of which a rubbing is preserved in Rikkyoku-an 栗棘庵, Tōfuku Temple 東福寺, Kyoto, Japan.

3 On Ryūsen’s maps, see Kaida Toshikazu, *Ryūsen zu to Sekisui zu: Edo jidai no besutoseraa Nihon chizu* (Tokyo: Ars Medica, 2017).

4 Marcia Yonemoto, *Mapping Early Modern Japan: Space, Place, and Culture in the Tokugawa Period, 1603–1868* (Berkeley: University of California Press, 2003), 32.

5 One exception in the case of Korea is a map of Korea made by Yun Tusŏ 尹斗緖 showing a more extensive route network. *Chido yech’an: Chosŏn chido 500-yŏn, konggan sigan in’gan ŭi iyagi* (Seoul: National Museum of Seoul, 2018), 46–47. One of the few Chinese examples is the ‘Haiyun tu’ 海運圖 (Sea transportation map) in Luo Hongxian’s 羅洪先 *Guang yutu* 廣輿圖 (Expanded terrestrial maps), *juan* 2. For a scan, see https://hdl.loc.gov/loc.asian/lcnclscd.2008623187.

6 Gari Ledyard, ‘Cartography in Korea,’ in *Cartography in the Traditional East and Southeast Asian Societies*, Vol. 2.2 of *The History of Cartography*, eds. J.B. Harley and David Woodward (Chicago: University of Chicago Press, 1994), 309.

7 On the Zheng He Map, see for example Roderich Ptak, ‘Selected Problems Concerning the “Zheng He Map”: Questions without Answers,’ *Journal of Asian History* 53, no. 2 (2019): 179–214. For a scan of the map contained in the military encyclopaedia *Wubei zhi* 武備志 (1621), see http://hdl.loc.gov/loc.gmd/g7821rm.gct00058.

8 Richard A. Pegg, ‘For the Record: Chinese Investiture Missions to Ryūkyū, 1404–1866,’ *Crossroads: An Interdisciplinary Journal of Asian Interactions* 20, no. 1–2 (2021): 1–27.

9 The Selden Map was discovered in the Bodleian Library, Oxford, in 2008, where it is still held. Since then a range of studies on the map have been published. In English, see, for example, Hongping Annie Nie, *The Selden Map of China. A New Understanding of the Ming Dynasty* (Oxford: Bodleian Libraries University of Oxford, 2019), and the literature cited therein. A scan of the map is available online: https://iiif.bodleian.ox.ac.uk/iiif/viewer/58b9518f-d5ea-4cb3-aa15-f42640c50ef3#?c=0&m=0&s=0&cv=0&r=0&xywh=-7409%2C-579%2C21708%2C11561.

10 On these screen maps, see Joseph F. Loh, ‘When Worlds Collide—Art, Cartography, and Japanese Nanban World Map Screens’ (PhD diss., Columbia University, 2013).

11 Kaida Toshikazu, *Zusetsu zōran: Edo jidai ni kankō sareta sekai chizu* (Tokyo: Ars Medica, 2019), 152–284; Richard A Pegg, *Cartographic Traditions in East Asian Maps* (Honolulu: MacLean Collection, University of Hawaiʻi Press, 2014), 35–42.

12 One exception of a Japanese mapmaker who kept the annotations on the European explorers is Takahashi Kageyasu 高橋景保 on his *Shintei bankoku zenzu* 新訂万國全圖 (1810). For a detailed study of this map, see Ninomiya Rikuo, *Takahashi Kageyasu to “Shintei bankoku zenzu”: Shin hakken to Arousumisu hōzu* (Sapporo: Hokkaidō shuppan kikaku sentā, 2007).

13 *Chido yech’an*, 46–47.

14 Richard A. Pegg, ‘A Chinese Map of the World Dated 1858,’ *Mapline* 125 (2016): 1–11; Bae Woo Sung, ‘Worldviews & Early Cartography,’ in *The Artistry of Early Korean Cartography*, trans. Choi Byonghyon (Larkspur: Tamal Vista Publications, 2008), 110–13.

15 See Jason Hubbard, *Japoniæ Insulæ. The Mapping of Japan. Historical Introduction and Cartobibliography of European Printed Maps of Japan to 1800* (Houten: Hes & De Graaf, 2012).

